# 2-[(1*H*-Benzimidazol-2-yl)sulfan­yl]-1-phenyl­ethanone

**DOI:** 10.1107/S1600536812028747

**Published:** 2012-06-30

**Authors:** Hatem A. Abdel-Aziz, Tze Shyang Chia, Hoong-Kun Fun

**Affiliations:** a Department of Pharmaceutical Chemistry, College of Pharmacy, King Saud University, PO Box 2457, Riyadh 11451, Saudi Arabia; b X-ray Crystallography Unit, School of Physics, Universiti Sains Malaysia, 11800 USM, Penang, Malaysia

## Abstract

The title compound, C_15_H_12_N_2_OS, adopts a twisted V-shape, with the S atom as the pivot. The benzimidazole ring system [maximum deviation = 0.015 (1) Å] makes a dihedral angle of 78.56 (7)° with the phenyl ring. The O atom of the ketone group is close to coplanar with its adjacent ring [O—C—C—C torsion angle = 11.0 (2)°]. In the crystal, mol­ecules are linked by N—H⋯N hydrogen bonds into an infinite chain along [001]. The crystal packing also features a C—H⋯π inter­action.

## Related literature

For a related structure. see: Abdel-Aziz *et al.* (2011 ▶). For the synthesis, see: D’Amico *et al.* (1964 ▶). For the stability of the temperature controller used in the data collection, see: Cosier & Glazer (1986 ▶). For standard bond lengths, see: Allen *et al.* (1987) ▶.
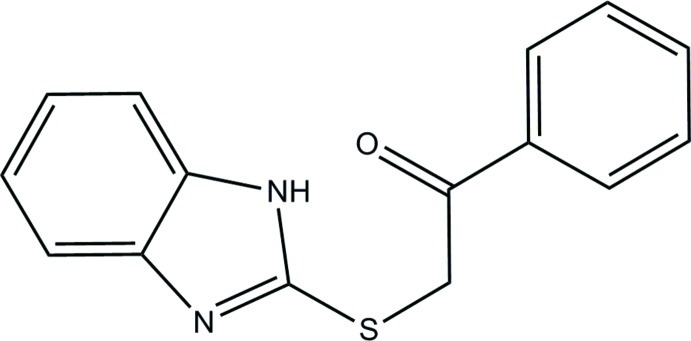


## Experimental

### 

#### Crystal data

C_15_H_12_N_2_OS
*M* *_r_* = 268.33
Monoclinic, 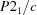

*a* = 14.7849 (13) Å
*b* = 9.2643 (8) Å
*c* = 9.7859 (8) Å
β = 106.792 (1)°
*V* = 1283.24 (19) Å^3^
*Z* = 4
Mo *K*α radiation
μ = 0.24 mm^−1^
*T* = 100 K
0.32 × 0.11 × 0.07 mm

#### Data collection

Bruker APEX DUO CCD diffractometer
Absorption correction: multi-scan (*SADABS*; Bruker, 2009 ▶) *T*_min_ = 0.927, *T*_max_ = 0.983
13644 measured reflections
3722 independent reflections
3094 reflections with *I* > 2σ(*I*)’
*R*_int_ = 0.027

#### Refinement

*R*[*F* ^2^ > 2σ(*F* ^2^)] = 0.041
*wR*(*F* ^2^) = 0.096
*S* = 1.05
3722 reflections
176 parameters
H atoms treated by a mixture of independent and constrained refinement
Δρ_max_ = 0.51 e Å^−3^
Δρ_min_ = −0.41 e Å^−3^

### 

Data collection: *APEX2* (Bruker, 2009 ▶); cell refinement: *SAINT* (Bruker, 2009 ▶); data reduction: *SAINT*; program(s) used to solve structure: *SHELXTL* (Sheldrick, 2008 ▶); program(s) used to refine structure: *SHELXTL*; molecular graphics: *SHELXTL*; software used to prepare material for publication: *SHELXTL* and *PLATON* (Spek, 2009 ▶).

## Supplementary Material

Crystal structure: contains datablock(s) global, I. DOI: 10.1107/S1600536812028747/hb6869sup1.cif

Structure factors: contains datablock(s) I. DOI: 10.1107/S1600536812028747/hb6869Isup2.hkl

Supplementary material file. DOI: 10.1107/S1600536812028747/hb6869Isup3.cml

Additional supplementary materials:  crystallographic information; 3D view; checkCIF report

## Figures and Tables

**Table 1:** Hydrogen-bond geometry (Å, °) *Cg*1 is the centroid of the N1/N2/C1/C6/C7 ring.

*D*—H⋯*A*	*D*—H	H⋯*A*	*D*⋯*A*	*D*—H⋯*A*
N2—H1*N*2⋯N1^i^	0.880 (19)	1.95 (2)	2.8250 (16)	175.8 (18)
C4—H4*A*⋯*Cg*1^ii^	0.93	2.82	3.4175 (15)	123

Symmetry codes: (i) 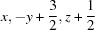; (ii) 
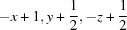
.

**Fig. 1.**
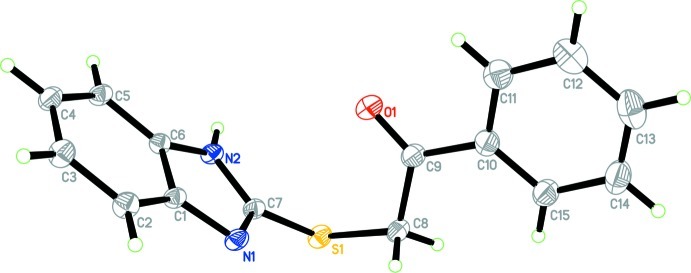
The molecular structure of the title compound with 50% probability displacement ellipsoids.

**Fig. 2.**
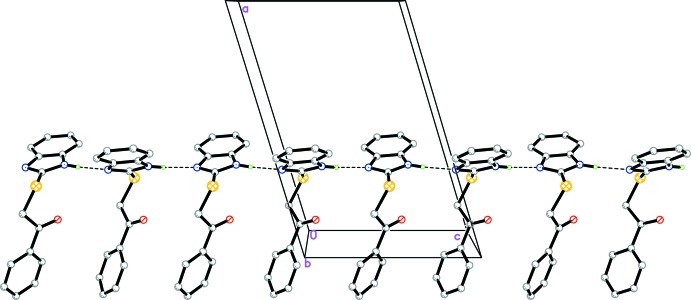
The crystal packing of the title compound. The dashed lines represent the hydrogen bonds. For clarity sake, hydrogen atoms not involved in hydrogen bonding have been omitted.

## supplementary crystallographic information

### Comment

In continuation to our reports on the chemistry and the biological activity of benzimidazoles (Abdel-Aziz *et al.*, 2011), we report herein the crystal structure of the title compound.

The asymmetric unit of the title compound is shown in Fig. 1. The molecule adopts a twisted V shape with S atom as the pivot, which is identical to a related structure (Abdel-Aziz *et al.*, 2011). The benzimidazole ring system (N1/N2/C1–C7) is essentially planar [maximum deviation = 0.015 (1) Å at atom C1] and makes a dihedral angle of 78.56 (7)° with the terminal C10–C15 benzene ring. The ketone group (C9═O1) is almost coplanar with the C10–C15 benzene ring as indicated by the O1—C9—C10—C11 torsion angle of 11.0 (2)°. Bond lengths (Allen *et al.*, 1987) and angles are within normal ranges.

In the crystal (Fig. 2), molecules are linked by N2—H1N2···N1 hydrogen bond into an infinite chain along the *c*-axis. The crystal packing is further stabilized by C—H···π interaction (Table 1), involving *Cg*1, which is the centroid of N1/N2/C1/C6/C7 ring.

### Experimental

The title compound was prepared by the reaction of 1*H*-benzo[*d*]imidazole-2-thiol and 2-bromo-1-phenylethanone in ethanol in the presence of potassium hydroxide (D'Amico *et al.*, 1964). Colourless needles were crystallised from ethanol solution.

### Refinement

The atom H1N2 was located in a difference fourier map and refined freely [N2—H1N2 = 0.880 (19) Å]. The remaining H atoms were positioned geometrically [C—H = 0.93 and 0.97 Å] and refined using a riding model with *U*_iso_(H) = 1.2*U*_eq_(C).

### Crystal data

**Table:**

C_15_H_12_N_2_OS	*F*(000) = 560
*M**_r_* = 268.33	*D*_x_ = 1.389 Mg m^−^^3^
Monoclinic, *P*2_1_/*c*	Mo *K*α radiation, λ = 0.71073 Å
Hall symbol: -P 2ybc	Cell parameters from 5435 reflections
*a* = 14.7849 (13) Å	θ = 2.6–30.0°
*b* = 9.2643 (8) Å	µ = 0.24 mm^−^^1^
*c* = 9.7859 (8) Å	*T* = 100 K
β = 106.792 (1)°	Needle, colourless
*V* = 1283.24 (19) Å^3^	0.32 × 0.11 × 0.07 mm
*Z* = 4	

### Data collection

**Table:**

Bruker APEX DUO CCD diffractometer	3722 independent reflections
Radiation source: fine-focus sealed tube	3094 reflections with *I* > 2σ(*I*)'
Graphite monochromator	*R*_int_ = 0.027
φ and ω scans	θ_max_ = 30.0°, θ_min_ = 1.4°
Absorption correction: multi-scan (*SADABS*; Bruker, 2009)	*h* = −20→20
*T*_min_ = 0.927, *T*_max_ = 0.983	*k* = −10→13
13644 measured reflections	*l* = −13→13

### Refinement

**Table:**

Refinement on *F*^2^	Primary atom site location: structure-invariant direct methods
Least-squares matrix: full	Secondary atom site location: difference Fourier map
*R*[*F*^2^ > 2σ(*F*^2^)] = 0.041	Hydrogen site location: inferred from neighbouring sites
*wR*(*F*^2^) = 0.096	H atoms treated by a mixture of independent and constrained refinement
*S* = 1.05	*w* = 1/[σ^2^(*F*_o_^2^) + (0.0361*P*)^2^ + 0.7649*P*] where *P* = (*F*_o_^2^ + 2*F*_c_^2^)/3
3722 reflections	(Δ/σ)_max_ = 0.001
176 parameters	Δρ_max_ = 0.51 e Å^−^^3^
0 restraints	Δρ_min_ = −0.41 e Å^−^^3^

### Special details

**Table:**

Experimental. The crystal was placed in the cold stream of an Oxford Cryosystems Cobra open-flow nitrogen cryostat (Cosier & Glazer, 1986) operating at 100.0 (1) K.
Geometry. All e.s.d.'s (except the e.s.d. in the dihedral angle between two l.s. planes) are estimated using the full covariance matrix. The cell e.s.d.'s are taken into account individually in the estimation of e.s.d.'s in distances, angles and torsion angles; correlations between e.s.d.'s in cell parameters are only used when they are defined by crystal symmetry. An approximate (isotropic) treatment of cell e.s.d.'s is used for estimating e.s.d.'s involving l.s. planes.
Refinement. Refinement of *F*^2^ against ALL reflections. The weighted *R*-factor *wR* and goodness of fit *S* are based on *F*^2^, conventional *R*-factors *R* are based on *F*, with *F* set to zero for negative *F*^2^. The threshold expression of *F*^2^ > σ(*F*^2^) is used only for calculating *R*-factors(gt) *etc*. and is not relevant to the choice of reflections for refinement. *R*-factors based on *F*^2^ are statistically about twice as large as those based on *F*, and *R*- factors based on ALL data will be even larger.

### Fractional atomic coordinates and isotropic or equivalent isotropic displacement parameters (Å^2^)

**Table:**

	*x*	*y*	*z*	*U*_iso_*/*U*_eq_	
S1	0.27768 (2)	0.54949 (4)	0.10069 (4)	0.02057 (9)	
O1	0.12453 (8)	0.75548 (13)	0.11153 (13)	0.0336 (3)	
N1	0.33134 (8)	0.80747 (12)	0.01048 (12)	0.0170 (2)	
N2	0.34377 (8)	0.79475 (12)	0.24453 (12)	0.0159 (2)	
C1	0.36703 (9)	0.93757 (14)	0.07565 (14)	0.0155 (2)	
C2	0.39488 (9)	1.06192 (15)	0.01768 (15)	0.0195 (3)	
H2A	0.3892	1.0686	−0.0793	0.023*	
C3	0.43124 (10)	1.17483 (15)	0.10989 (15)	0.0209 (3)	
H3A	0.4504	1.2587	0.0739	0.025*	
C4	0.43994 (9)	1.16595 (15)	0.25666 (15)	0.0198 (3)	
H4A	0.4649	1.2439	0.3154	0.024*	
C5	0.41217 (9)	1.04368 (14)	0.31575 (14)	0.0179 (3)	
H5A	0.4175	1.0378	0.4126	0.021*	
C6	0.37587 (8)	0.93011 (13)	0.22273 (14)	0.0149 (2)	
C7	0.31869 (9)	0.72789 (14)	0.11564 (14)	0.0163 (2)	
C8	0.16524 (10)	0.57212 (15)	−0.03317 (16)	0.0224 (3)	
H8A	0.1767	0.6038	−0.1212	0.027*	
H8B	0.1340	0.4790	−0.0513	0.027*	
C9	0.09888 (10)	0.67930 (16)	0.00635 (16)	0.0229 (3)	
C10	0.00060 (10)	0.69134 (16)	−0.09324 (16)	0.0224 (3)	
C11	−0.05419 (11)	0.80839 (17)	−0.07551 (18)	0.0277 (3)	
H11A	−0.0292	0.8754	−0.0038	0.033*	
C12	−0.14568 (12)	0.82596 (19)	−0.1637 (2)	0.0335 (4)	
H12A	−0.1814	0.9051	−0.1520	0.040*	
C13	−0.18359 (11)	0.7248 (2)	−0.26939 (19)	0.0340 (4)	
H13A	−0.2448	0.7364	−0.3290	0.041*	
C14	−0.13037 (11)	0.6066 (2)	−0.28627 (18)	0.0327 (4)	
H14A	−0.1565	0.5380	−0.3559	0.039*	
C15	−0.03793 (11)	0.58989 (18)	−0.19947 (17)	0.0271 (3)	
H15A	−0.0020	0.5114	−0.2123	0.033*	
H1N2	0.3370 (13)	0.761 (2)	0.325 (2)	0.030 (5)*	

### Atomic displacement parameters (Å^2^)

**Table:**

	*U*^11^	*U*^22^	*U*^33^	*U*^12^	*U*^13^	*U*^23^
S1	0.02627 (17)	0.01719 (15)	0.01866 (18)	−0.00276 (12)	0.00714 (13)	−0.00024 (13)
O1	0.0277 (5)	0.0396 (6)	0.0327 (7)	−0.0016 (5)	0.0075 (5)	−0.0181 (5)
N1	0.0216 (5)	0.0182 (5)	0.0112 (5)	−0.0013 (4)	0.0048 (4)	−0.0010 (4)
N2	0.0204 (5)	0.0178 (5)	0.0098 (5)	−0.0002 (4)	0.0050 (4)	0.0009 (4)
C1	0.0172 (5)	0.0176 (6)	0.0114 (6)	0.0005 (4)	0.0036 (4)	−0.0004 (5)
C2	0.0236 (6)	0.0214 (6)	0.0132 (6)	−0.0005 (5)	0.0048 (5)	0.0026 (5)
C3	0.0249 (6)	0.0177 (6)	0.0199 (7)	−0.0014 (5)	0.0062 (5)	0.0023 (5)
C4	0.0213 (6)	0.0187 (6)	0.0185 (7)	−0.0007 (5)	0.0044 (5)	−0.0042 (5)
C5	0.0205 (6)	0.0209 (6)	0.0116 (6)	0.0007 (5)	0.0037 (5)	−0.0018 (5)
C6	0.0154 (5)	0.0165 (5)	0.0126 (6)	0.0012 (4)	0.0038 (4)	0.0011 (5)
C7	0.0182 (6)	0.0180 (6)	0.0128 (6)	0.0003 (5)	0.0046 (5)	−0.0003 (5)
C8	0.0242 (6)	0.0234 (6)	0.0202 (7)	−0.0050 (5)	0.0072 (5)	−0.0067 (6)
C9	0.0242 (6)	0.0237 (6)	0.0222 (7)	−0.0046 (5)	0.0093 (6)	−0.0047 (6)
C10	0.0237 (6)	0.0253 (7)	0.0200 (7)	−0.0053 (5)	0.0091 (5)	0.0006 (6)
C11	0.0300 (7)	0.0249 (7)	0.0291 (8)	−0.0027 (6)	0.0101 (6)	0.0002 (6)
C12	0.0302 (8)	0.0341 (8)	0.0376 (10)	0.0035 (7)	0.0120 (7)	0.0092 (7)
C13	0.0247 (7)	0.0469 (10)	0.0290 (9)	−0.0046 (7)	0.0056 (6)	0.0120 (8)
C14	0.0301 (7)	0.0440 (9)	0.0229 (8)	−0.0120 (7)	0.0056 (6)	−0.0025 (7)
C15	0.0271 (7)	0.0316 (8)	0.0233 (8)	−0.0059 (6)	0.0084 (6)	−0.0044 (6)

### Geometric parameters (Å, º)

**Table:**

S1—C7	1.7519 (13)	C5—H5A	0.9300
S1—C8	1.8070 (15)	C8—C9	1.5222 (19)
O1—C9	1.2148 (18)	C8—H8A	0.9700
N1—C7	1.3224 (17)	C8—H8B	0.9700
N1—C1	1.3941 (16)	C9—C10	1.502 (2)
N2—C7	1.3571 (17)	C10—C11	1.394 (2)
N2—C6	1.3790 (16)	C10—C15	1.395 (2)
N2—H1N2	0.880 (19)	C11—C12	1.388 (2)
C1—C2	1.3977 (18)	C11—H11A	0.9300
C1—C6	1.4088 (18)	C12—C13	1.388 (3)
C2—C3	1.3846 (19)	C12—H12A	0.9300
C2—H2A	0.9300	C13—C14	1.386 (3)
C3—C4	1.407 (2)	C13—H13A	0.9300
C3—H3A	0.9300	C14—C15	1.394 (2)
C4—C5	1.3869 (19)	C14—H14A	0.9300
C4—H4A	0.9300	C15—H15A	0.9300
C5—C6	1.3934 (18)		
			
C7—S1—C8	100.07 (6)	C9—C8—H8A	108.6
C7—N1—C1	104.24 (11)	S1—C8—H8A	108.6
C7—N2—C6	106.56 (11)	C9—C8—H8B	108.6
C7—N2—H1N2	126.9 (12)	S1—C8—H8B	108.6
C6—N2—H1N2	126.3 (12)	H8A—C8—H8B	107.6
N1—C1—C2	130.08 (12)	O1—C9—C10	120.88 (13)
N1—C1—C6	109.64 (11)	O1—C9—C8	121.87 (13)
C2—C1—C6	120.26 (12)	C10—C9—C8	117.22 (12)
C3—C2—C1	117.56 (12)	C11—C10—C15	119.34 (14)
C3—C2—H2A	121.2	C11—C10—C9	117.71 (13)
C1—C2—H2A	121.2	C15—C10—C9	122.94 (13)
C2—C3—C4	121.66 (12)	C12—C11—C10	120.72 (15)
C2—C3—H3A	119.2	C12—C11—H11A	119.6
C4—C3—H3A	119.2	C10—C11—H11A	119.6
C5—C4—C3	121.51 (13)	C11—C12—C13	119.69 (16)
C5—C4—H4A	119.2	C11—C12—H12A	120.2
C3—C4—H4A	119.2	C13—C12—H12A	120.2
C4—C5—C6	116.68 (12)	C14—C13—C12	120.09 (15)
C4—C5—H5A	121.7	C14—C13—H13A	120.0
C6—C5—H5A	121.7	C12—C13—H13A	120.0
N2—C6—C5	132.10 (12)	C13—C14—C15	120.36 (16)
N2—C6—C1	105.56 (11)	C13—C14—H14A	119.8
C5—C6—C1	122.34 (12)	C15—C14—H14A	119.8
N1—C7—N2	114.00 (12)	C14—C15—C10	119.79 (15)
N1—C7—S1	125.75 (10)	C14—C15—H15A	120.1
N2—C7—S1	120.22 (10)	C10—C15—H15A	120.1
C9—C8—S1	114.65 (10)		
			
C7—N1—C1—C2	179.02 (13)	C6—N2—C7—S1	177.86 (9)
C7—N1—C1—C6	0.78 (14)	C8—S1—C7—N1	−57.99 (13)
N1—C1—C2—C3	−177.79 (13)	C8—S1—C7—N2	124.34 (11)
C6—C1—C2—C3	0.30 (19)	C7—S1—C8—C9	−57.82 (11)
C1—C2—C3—C4	−0.1 (2)	S1—C8—C9—O1	8.48 (19)
C2—C3—C4—C5	−0.2 (2)	S1—C8—C9—C10	−173.67 (10)
C3—C4—C5—C6	0.38 (19)	O1—C9—C10—C11	11.0 (2)
C7—N2—C6—C5	−178.49 (13)	C8—C9—C10—C11	−166.86 (13)
C7—N2—C6—C1	0.55 (13)	O1—C9—C10—C15	−167.78 (15)
C4—C5—C6—N2	178.68 (13)	C8—C9—C10—C15	14.4 (2)
C4—C5—C6—C1	−0.22 (19)	C15—C10—C11—C12	−1.0 (2)
N1—C1—C6—N2	−0.83 (14)	C9—C10—C11—C12	−179.88 (14)
C2—C1—C6—N2	−179.28 (11)	C10—C11—C12—C13	0.9 (2)
N1—C1—C6—C5	178.32 (11)	C11—C12—C13—C14	0.3 (2)
C2—C1—C6—C5	−0.12 (19)	C12—C13—C14—C15	−1.4 (2)
C1—N1—C7—N2	−0.44 (15)	C13—C14—C15—C10	1.2 (2)
C1—N1—C7—S1	−178.24 (10)	C11—C10—C15—C14	0.0 (2)
C6—N2—C7—N1	−0.07 (15)	C9—C10—C15—C14	178.74 (14)

### Hydrogen-bond geometry (Å, º)

*Cg*1 is the centroid of the N1/N2/C1/C6/C7 ring.

**Table:**

*D*—H···*A*	*D*—H	H···*A*	*D*···*A*	*D*—H···*A*
N2—H1*N*2···N1^i^	0.880 (19)	1.95 (2)	2.8250 (16)	175.8 (18)
C4—H4*A*···*Cg*1^ii^	0.93	2.82	3.4175 (15)	123

Symmetry codes: (i) *x*, −*y*+3/2, *z*+1/2; (ii) −*x*+1, *y*+1/2, −*z*+1/2.
